# A four year longitudinal sero-epidemiology study of *Neospora caninum *in adult cattle from 114 cattle herds in south west England: Associations with age, herd and dam-offspring pairs

**DOI:** 10.1186/1746-6148-4-35

**Published:** 2008-09-15

**Authors:** Kerry A Woodbine, Graham F Medley, Stephen J Moore, Ana Ramirez-Villaescusa, Sam Mason, Laura E Green

**Affiliations:** 1 Department of Biological Science, University of Warwick, Coventry, CV4 7AL, UK

## Abstract

**Background:**

Neosporosis caused by the protozoan parasite *Neospora caninum*, is an economically important cause of abortion, stillbirth, low milk yield, reduced weight gain and premature culling in cattle. Consequently, a seroepidemiological study of *N. caninum *antibodies was conducted in England with 29,782 samples of blood taken from 15,736 cattle from 114 herds visited on three occasions at yearly intervals. Herds were categorised into lower (< 10%) and higher (≥ 10%) median herd seroprevalence. Hierarchical models were run to investigate associations between the sample to positive (S/P) ratio and herd and cattle factors.

**Results:**

Ninety-four percent of herds had at least one seropositive cow; 12.9% of adult cattle had at least one seropositive test. Approximately 90% of herds were seropositive at all visits; 9 herds (8%) changed serological status between visits. The median *N. caninum *seroprevalence in positive herds was 10% (range 0.4% to 58.8%). There was a positive association between the serostatus of offspring and dams that were ever seropositive. In the hierarchical model of low seroprevalence herds there was no significant association between S/P ratio and cattle age. There was a significantly lower S/P ratio in cattle in herds that were totally restocked after the foot-and-mouth epidemic of 2001 compared with those from continuously stocked herds and cattle purchased into these herds had a higher S/P ratio than homebred cattle. In the model of high seroprevalence herds the S/P ratio increased with cattle age, but was not associated with restocking or cattle origin.

**Conclusion:**

There were no strong temporal changes in herd seroprevalence of *N. caninum *but 90% of herds had some seropositive cattle over this time period. Vertical transmission from seropositive dams appeared to occur in all herds. In herds with a high seroprevalence the increasing S/P ratio in 2–4 year old cattle is suggestive of exposure to *N. caninum*: horizontal transmission between adult cattle, infection from a local source or recrudescence and abortions. Between-herd movements of infected cattle enhance the spread of *N. caninum*, particularly into low seroprevalence herds. Some restocked herds had little exposure to *N. caninum*, while in others infection had spread in the time since restocking.

## Background

*Neospora caninum *is an apicomplexan protozoan parasite that has a worldwide distribution. In the UK, approximately 12.5% of all cattle abortions were attributed *N. caninum *in 1997 [1]. Neosporosis also causes stillbirths, low milk yield [2], reduced weight gain [3] and premature culling [4,5] and is therefore responsible for considerable economic loss [6].

Dogs [7] and coyotes [8] are recognised as definitive hosts for *N. caninum*, while cattle and several other species, such as, deer, horses, water buffalo, goats and sheep act as possible intermediate hosts [9,10]. The *N. caninum *two-host life cycle has three infectious stages: sporozoites in oocysts, rapidly developing tachyzoites, and bradyzoites in tissue cysts. Unsporulated oocysts are excreted in the faeces of the definitive host and sporulated oocysts are ingested by intermediate hosts in contaminated food, water or soil. Sporozoites are then released and differentiate to tachyzoites then to bradyzoites which form tissue cysts. The definitive host acquires infection by ingesting tissue containing these cysts.

Cattle and definitive hosts can be infected by vertical transmission when tachyzoites cross the placenta and infect the foetus [11]. This can occur in consecutive pregnancies and so infection can persist through many generations [12-14]. Vertical transmission is considered the predominant route of transmission in cattle [15-17], with an efficacy of up to 95.2% in chronically infected cows [18]. However, for *N. caninum *to be introduced into, and persist in, a susceptible population, an infected cow must be introduced and subsequently transmit infection to her daughters vertically and to other cattle in the population horizontally, possibly via the definitive host [19]. There are several reports of horizontal transmission of *N. caninum *[18,20,16], and in one study the probability that horizontal transmission increased as herd seroprevalence increased was reported [21].

The percentage of seropositive cattle within herds varies by country, region and herd, and depends in part on the type of serological test performed and the test cut-off used [22]. In one study, 17.1% of 4,295 cattle from 14 British dairy herds were seropositive with a herd-specific prevalence ranging from 7.3% to 44.8% [23]. In these 14 herds there was no association between seroprevalence and herd size or cattle age. The seroprevalence of *N. caninum *in 418 dairy cows that calved normally was 6% (95% CI, 4% – 8%) compared with 18% in 633 recently-aborted cattle (95% CI, 15% – 21%) [1]. In similar case control studies in Northern Ireland and Scotland, the seroprevalence of *N. caninum *in cows that calved normally were 3% and 1%, and in cows that aborted the seroprevalences were 12.6% and 9% respectively [24,25].

In a large-scale inter-country study using in-house and commercial ELISA kits, *N. caninum *was present in 16%, 49%, 63% and 76% of dairy herds in Sweden, Germany, Spain and the Netherlands respectively. The prevalence of seropositive suckler herds was 41% in Germany, 46% in Spain, and 61% in the Netherlands. Cattle seroprevalence within herds ranged from 0.5% in Sweden to 16.2% in Spain [26].

There is no known method for control of neosporosis. A licensed vaccine to prevent *N. caninum *abortion in cattle is not available in the UK. Current advice for control of neosporosis is therefore based on improving farm management practices. This includes avoiding exposure of cattle to dog faeces, prompt removal of aborted foetuses and dead calves, culling of cows that repeatedly abort and quarantine and testing of replacement cattle [27,28].

In this paper we present the patterns and temporal changes *N. caninum *antibodies from a 4-year cohort study of 15,736 cattle in 114 English cattle herds, and the associations between the continuous outcome the sample to positive (S/P) ratio and cattle age, whether purchased or homebred and mean herd seroprevalence.

## Methods

### Source of data

#### Farms

The data used in this study came from a 4-year cohort study of 114 cattle (dairy and suckler) herds in south west England that took place from 2002 – 2006. All farms were situated in areas within the Randomised Badger Culling Trial (RBCT) that was conducted in England from 1998 – 2005 [29] and in an area where some herds were restocked (i.e. completely depopulated and subsequently restocked) after the 2001 foot-and-mouth disease (FMD) epidemic [30]. The farms in this study were a convenience selected sub-sample of those in the RBCT. They were cattle herds with breeding cattle that could be sampled on up to 3 occasions, with farmers that permitted samples of blood to be taken from their cattle.

#### Serum samples

Up to three routine visits were made to each farm, approximately one year apart, to collect samples of blood. Samples (up to 10 ml) were collected under Home Office licence (that is, sample collection was authorised under the Animals in Scientific Procedures legislation) from all accessible cattle ≥ 2 years of age. A subset of herds (n = 15) were re-visited a fourth time and blood samples were taken from cattle of all ages. These herds were re-visited either to re-test individual cattle to confirm whether they were persistently infected (PI) with bovine viral diarrhoea virus (BVDV) or to sample the whole herd (including young stock) after a BVDV PI had been detected in the adult herd. It is worth noting that additional visits took place to confirm BVDV, not *N. caninum *status. Four herds had a whole herd test instead of a routine third visit because a BVDV antigen positive sample was detected before this third visit. Blood samples were centrifuged at the University of Warwick at 3220 g for 15 minutes and serum was removed, frozen and stored at -20°C until they were analysed.

### Questionnaire

Farmers were interviewed between 17^th ^June 2003 and the end of February 2004 using a questionnaire that comprised primarily closed and semi-closed questions to obtain information about clinical *N. caninum *disease (including abortion rates and if the veterinarian had diagnosed Neosporosis). Participation in the questionnaire was > 95%.

#### Matching data with external databases

Cattle ear tag or freeze brand numbers were recorded during each visit; when a freeze brand was taken the farmer provided a list linking the freeze brand and ear tag. The ear tag was matched with information from the Cattle Tracing System (CTS). Less than one percent (371) of ear tag numbers could not be matched with the CTS data. Ninety percent of these cases were because the same identifier had been recorded twice; other errors were that the cattle did not have a freeze brand or ear tag. The ear tag number also linked to the British Cattle Movement Service (BCMS) database where data on date of birth, origin (whether it was homebred or purchased), breed, dam and sex were sourced. From 2001 it became compulsory to record all cattle birth dates. Herd size was estimated from the cattle tuberculosis testing data (VetNet database).

Laboratory results, questionnaire data and external data were entered into a relational database (PostgreSQL, PostgreSQL Global Development Group) using Microsoft Access (Microsoft Corp. US) as a front end. All data were checked for errors and data were re-entered where errors were detected.

#### Serological test and interpretation

All enzyme linked immunosorbent assay (ELISA) testing was done at the University of Warwick, England. The HerdChek Anti-*Neospora caninum *antibody kit (IDEXX), an ELISA for the detection of antibody against *N. caninum*, was used to test serum for presence of *N. caninum *antibody. The tests were performed according to the kit instructions. An internal quality control sample was included on every plate to control for batch to batch variation. All samples were run in duplicate.

Positive samples were re-tested when the duplicates were more than 0.25 optical density (OD) units apart or when the OD units of the positive controls were more than 0.2 OD units apart. The sensitivity and specificity of this serological assay has been reported to be 100% (95% CI, 100.0% – 100.0%) and 99.7% (95% CI, 99.1% – 100.0%) respectively [31], and 93% and 94% respectively [22]. The level of antibodies to *N. caninum *was expressed as the sample to positive control (S/P) ratio using the calculation:

$$S/P=\frac{OD_{Sample}-OD_{NegativeControl}}{OD_{PositiveControl}-OD_{NegativeControl}}$$

A sample was positive when the S/P ratio ≥ 0.5 and negative when the S/P ratio < 0.5. The S/P ratio took the values -0.29 to 6.27.

#### Datasets used

Two datasets were used. Dataset A (29,782 samples, 15,736 cattle, 114 herds) with all serological results for all cattle from all visits, and Dataset B (26,437 samples, 13,942 cattle, 114 herds) with serological results from cattle ≥ 2 years of age from the three planned herd visits and from the four whole herd visits that replaced the third visit. Hence, Dataset B is a subset of Dataset A.

### Outcome variables

A herd was defined as seropositive when at least one cow in the herd tested positive to *N. caninum *on one occasion.

A cow was defined as *N. caninum *seropositive when at least one of its samples was positive.

The herd seroprevalence of *N. caninum *was calculated from the number of seropositive cattle divided by the total number of cattle tested at the visit.

The median herd seroprevalence was 10%. Herds were divided at the median into < 10%, (n = 60 herds) and ≥ 10%, (n = 54) seroprevalence based on their mean herd seroprevalence from the routine visits.

The S/P ratio was used as the continuous outcome measure when modelling antibody levels (rather than the dichotomous seroprevalence).

### Statistical analysis

Data were screened using univariate analysis and then multilevel models were developed using the continuous outcome variable S/P ratio [32]. Dataset B was used to avoid any potential bias from the non-routine visits. Two multilevel models were run, one using herds with a seroprevalence of < 10% and another using herds with a seroprevalence ≥ 10%. There were three hierarchical levels in the models: routine visits (level 1), clustered by cattle (level 2), and herd (level 3) to control for clustering at the cow and herd levels. The multivariable model was built using both manual forward selection and backwards elimination. Variables with a p-value of < 0.20 in the univariate analysis were tested in the final models. Univariate variables were run including all cattle in Dataset B and excluding cattle with missing data. All analyses were done using MLwiN (version 2.1, Centre for Multilevel Modelling, London, UK).

Each model took the form:

*S*/*P*_*ijk *_= *βX*_0 _+ *βX*_*k *_+ *βX*_*jk *_+ *βX*_*ijk *_+ *ν*_*k*_+*u*_*jk *_+ *e*_*ijk*_

where *S*/*P*_*ijk *_is the value of the outcome of the *i*th visit (sample) from the *j*th cow in the *k*th herd. *βX*_0 _is the intercept, *βX *is a series of vectors of fixed effects varying at herd (*k*), cattle (*jk*) and visit (*ijk*), *ν*_*k *_+ *u*_*jk *_are the variances at the herd and cattle levels respectively, and *e*_*ijk *_is the residual variance. The adequacy of the final model and the assumption of normality were inspected by plotting the residuals in ascending order with their 95% confidence limit [32]. All explanatory variables were compared for correlations by chi-squared analysis. The models were re-run as logistic binomial models with the outcome seropositive or seronegative, to compare significant variables.

### The explanatory variables

The fixed effects in Table 1 were tested in the multivariable model. The variables triplet code (i.e. what form of badger control was applied on that farm during the RBCT), restocking status and farm location were forced into the model because they formed part of the study design.

**Table 1:** Definitions for explanatory variables tested in the hierarchical model.

Explanatory variable	Variable defined
Triplet code	Treatment in the Random Badger Culling Trial (RBCT) (Bourne et al., 2007)
	Proactive – all badgers culled
	Reactive – badgers culled in response to tuberculosis herd breakdown
	Survey – no badgers culled
FMD restocked or continuously stocked	Herd was totally depopulated or herd was not totally depopulated in 2001 due to the foot-and-mouth (FMD) epidemic
Geographical area	Farm location
	Area A – (Gloucestershire Herefordshire/Worcestershire)
	Area B – (North East Devon South Somerset)
	Area C – (North West Devon North East Cornwall)
Log (mean herd size)	The log mean number of cattle present in the herd during study period logged from the VetNet database
Cattle sex	Cattle were female or male taken from the British Cattle Movement System
Origin of replacement cattle	Homebred (tested in natal herd) Purchased (tested in different from natal herd)
Mean herd *N caninum *seroprevalence	Lower or higher than the overall median herd *N caninum *seroprevalence of 10%
Cattle age (years)	In yearly intervals from 2 years to 10+ years old

## Results

### Descriptive statistics

#### Study population

There were 107 herds (93.9%, 95% CI, 92.3% – 95.5%) with at least one *N. caninum *seropositive cow on one occasion (Table 2). Three herds were positive at the first and third visits but negative at the second visit, three herds were positive at the first but negative at the second and third visit, two herds were negative at the first visit but positive at the second and third visit and one herd was positive at the first and second visits but negative at the third visit. Twenty-seven percent (31/114) of herds had a mean *N. caninum *seroprevalence between 5 and 10%, and 2.6% (3/114) of herds had a mean *N. caninum *seroprevalence of greater than 40%. These were 2 suckler herds and 1 dairy herd which were not depopulated after the FMD and were all situated in Gloucestershire. All three herds had a mean herd size > 100 cattle during the study period, with 32 to 55 cattle sampled at each visit, and had no unusual cattle age structure (i.e. herds did not consist of all old or young cattle). From the data obtained these herds did not differ greatly from the other herds in the study. For example, there was no statistical difference between the median herd size for these herds and the other herds (*U *= 182.5; *P *< 0.05, Mann-Whitney *U*-test). Approximately 12.9% cattle tested positive at least once. A total of 12,139 cattle (87.1%, 95% CI, 86.6% – 87.6%) were always test negative, and 1,127 (8.1%, 95% CI, 7.6% – 8.6%) were always test positive. A total of 676 cattle (4.8%) had different test results on two occasions: 213 cattle tested positive and then negative and 338 cattle tested negative then positive. 0.9% of cattle (125 cattle) changed serological status twice: 19 cattle tested seropositive, seronegative then seropositive and 106 cattle tested seronegative, seropositive and then seronegative.

**Table 2:** Number and percentage *N caninum *seropositive cattle and herds by visit types and the herd *N caninum *seroprevalence for the positive herds (Dataset B – 26,437 samples, 13,942 cattle, 114 herds, only cattle ≥ 2 years old for regular visits in the 4-year study period).

Visit identification	Herds			Cattle			Within herd *N. caninum *seroprevalence for the positive herds only			
	No. tested	No. pos	% pos	No. tested	No. pos	% pos	Median	Range	25^th ^quartile	75^th ^quartile

1^st ^routine visit	114	104	91.2	9963	1027	10.3	10.2	0.8 – 52.7	7.3	14.3
2^nd ^routine visit	102	91	89.2	8979	941	10.5	9.6	0.4 – 58.8	5.8	16.8
3^rd ^routine visit	96	87	90.6	8580	784	9.1	8.7	0.7 – 58.5	5.3	15.2
3^rd ^routine with whole herd visit	4	4	100.0	1135	139	12.2	18.8	5.6 – 27.3	14.2	23.6
Overall	114	107	93.9	15736	2039	12.9				

There were 9963, 8979 and 8580 samples respectively from the 114 herds visited once, 102 visited twice and 96 visited three times. An additional 1,135 samples were collected when four whole herd visits replaced the routine third visit (i.e. extra samples taken from young stock < 2 years).

If the 99.7% specificity of the kit is taken into account, there were in total 3,089 positive results during the whole of the study, approximately 9 cattle tests during the study were false positives, and if 94% specificity [22] estimate is used there would be 185 false positive cattle tests.

The mean herd size ranged from 3 to 847, and the number of adult cattle sampled at each routine visit ranged from 2 to 578. The average herd size was highly correlated with the number of adult cattle sampled. There was no association between herd size or the number of adult cattle sampled and the herd seroprevalence to *N. caninum*.

For 3,907 (24.8%) cattle there were no data on their origin because they were born before 2001. In the 11,181 cattle with data, a crude analysis indicated that purchased cattle were more likely to be *N. caninum *antibody seropositive (9.3%) than homebred cattle (6.5%). The trend was for the mean *N. caninum *S/P ratio to increase with time from purchase in cattle purchased into herds with a mean *N. caninum *seroprevalence ≥ 10% (0.36 for cattle that have been in herd less than a year to 0.58 for cattle that have been in herd for 4–5 years). However, herds with a mean *N. caninum *seroprevalence < 10% remained relatively constant (0.22 for cattle that have been in herd less than a year to 0.20 for cattle that have been in herd for 4–5 years).

Two (1.8%) herds had clinical signs of abortion, possibly attributable to neosporosis according to the farmer. The mean herd seroprevalences for these two herds were 29.3% and 10.8% respectively. A further fourteen farmers (12.7%) reported abortion in their cattle but the cause was not defined by the farmers. The mean herd seroprevalence for *N. caninum *for the 14 herds with reported cattle abortions was 9.1% (range: 2.6 – 20.0).

#### Horizontal transmission – case studies of individual herds

**Figure 1:**
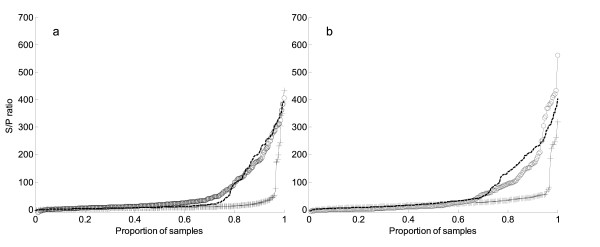
***N. caninum* S/P ratio against proportion of samples (positivity ramps) for two different herds in the study that had an outbreak between visit one and visit two.** Black crosses and line represents first routine visit black circles and line represents second routine visit and the black line represents the third routine visit for each herd (Dataset B – 26,437 samples, 13,942 cattle, 114 herds, only cattle ≥ 2 years old for regular visits in the 4-year study period).

In two herds (Figures 1a and 1b) a large proportion of cattle seroconverted between the first and second visits. This was unique for these two herds. In both cases, there were a small proportion of positive cattle at the first visit and an increased proportion by the second visit. For the first of these herds (Figure 1a) the pattern did not change between the second and third visit. These two herds were chosen because of the clear change in pattern between visits, and differences seen between the herds not because they had clinical disease.

#### Dam-offspring interactions (vertical transmission)

**Figure 2:**
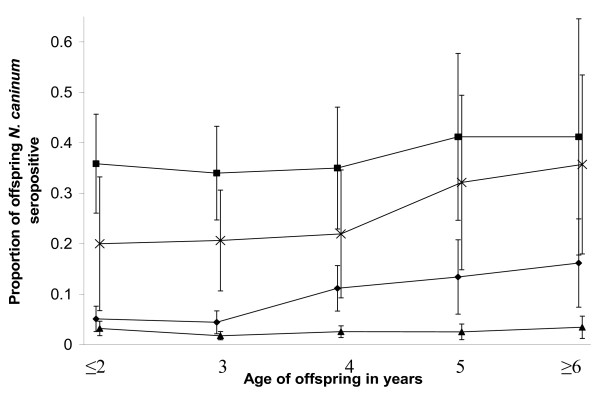
**The proportion of offspring *N. caninum *seropositive by age at testing, dam *N. caninum *status and herd mean seroprevalence < 10% or ≥ 10%, 95% confidence intervals included (Dataset A – 29,782 samples, 15,736 cattle, 114 herds, all cattle for the whole 4-year study period).** Squares represent dam always positive in herd with mean seroprevalence ≥ 10%, crosses represent dam always positive in herd with mean seroprevalence < 10%, dots represent dam always negative in herd with mean seroprevalence ≥ 10% and triangles represent dam always negative in herd with mean seroprevalence < 10%.

Offspring from dams that were *N. caninum *seropositive before calving were statistically more likely to be *N. caninum *seropositive than those born to dams negative during pregnancy (*T *= 2, *P *< 0.05, Wilcoxon's test for matched pairs). There was a trend for offspring from dams that were always *N. caninum *seropositive were also more likely to be *N. caninum *seropositive whatever the herd seroprevalence (Figure 2).

There was also a trend that offspring born to seropositive dams were more likely to be seropositive as age at sampling increased, especially after 4 years of age (Figure 2). This was also observed in offspring born to seronegative dams in herds with a mean herd *N. caninum *seroprevalence ≥ 10% but not for offspring born to seronegative dams in herds with a mean seroprevalence < 10%. There was also a significant statistical difference between the median *N. caninum *S/P ratio in all calves born from dams ≤ 5 years of age compared with those born to dams > 5 years of age regardless of herd seroprevalence or dam status (*T *= 8, *P *< 0.05, Wilcoxon's test for matched pairs), with the calves born from dams > 5 years of age having a higher median S/P ratio.

Using Dataset B, the mean S/P ratio were visually (but not significantly) lower during the first five months of pregnancy (first trimester) then rose during late pregnancy in dams that were seropositive before calving. This pattern was not observed in pregnant dams that were seronegative to *N. caninum *before calving.

#### Age-related *N. caninum *seroprevalence and seroconversion

**Figure 3:**
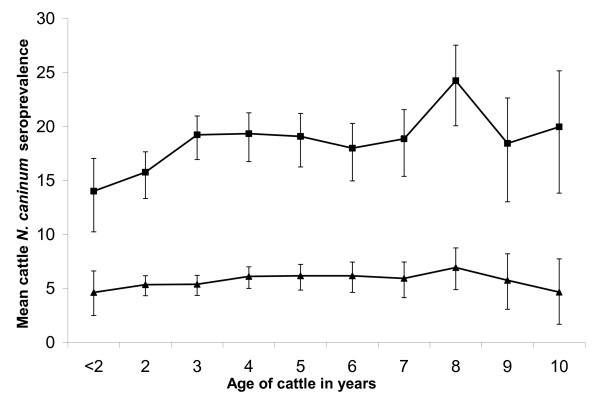
Age-specific *N. caninum *antibody seroprevalence for cattle ≥ 2 years of age sampled at the three routine herd visits (Dataset B – 26,437 samples, 13,942 cattle, 114 herds, only cattle ≥ 2 years old for regular visits in the 4-year study period) by herd seroprevalence to *N. caninum *< 10% and ≥ 10%. Squares represent cattle from herds with a mean seroprevalence < 10% and triangles represent cattle from herds with a mean seroprevalence ≥ 10%.

In herds with a mean *N. caninum *seroprevalence ≥ 10%, there was an increase in seroprevalence between 2 and 4 years of age. The seroprevalence of antibodies against *N. caninum *did not increase with age in herds with a mean *N. caninum *seroprevalence < 10%, where there was a constant seroprevalence of approximately 5% (Figure 3). In total, there were 235 cattle which had tested positive when > 3 years of age that had been sampled when ≤ 3 years of age. Of these, 84 cattle always tested negative until they were > 3 years of age, indicating possible horizontal transmission, rather than recrudescence. A further 25 of the 235 cattle tested both negative and positive, and 126 cattle always tested positive when ≤ 3 years of age.

#### Univariate analysis

Results from the univariate analysis are presented in Table 3. Farm location, age and purchased cattle were significantly related to the S/P ratio. Cattle origin contained the majority of missing data, when all cattle with missing data were excluded from the univariate analysis there was no evidence that the missing data was related to any of the remaining variables, therefore was random for the outcome.

**Table 3:** Univariate analysis of the fixed effects associated with *N. caninum *antibody S/P ratio, (Dataset B – 26,437 samples, 13,942 cattle, 114 herds, only cattle ≥ 2 years old for regular visits in the 4-year study period).

		**All data**			**Cattle with date of birth omitted data**		
**Variable**	**Category**	**Coef**	**SE^b^**	**P value**	**Coef**	**SE^b^**	**P value**

Triplet code^*a*^	Reactive						
	Proactive	-0.122	0.051	0.02	-0.084	0.049	0.09
	Survey	-0.097	0.051	0.06	-0.074	0.049	0.13
Restocked	No						
	Yes	-0.043	0.052	0.41	-0.026	0.050	0.60
Farm location	Area A						
	Area B	-0.153	0.045	< 0.01	-0.136	0.043	< 0.01
	Area C	-0.086	0.076	0.26	-0.035	0.074	0.64
Log (herd size)		-0.085	0.059	0.15	-0.079	0.059	0.18
Cattle sex	Female						
	Male	-0.075	0.052	0.15	-0.065	0.056	0.25
Replacement cattle	Homebred						
	Purchased	0.078	0.018	< 0.01	0.078	0.018	< 0.01
Mean herd seroprevalence	< 10%						
	≥ 10%	0.294	0.031	< 0.01	0.274	0.030	< 0.01
Age (years)	2						
	3	0.022	0.014	< 0.01	0.021	0.014	0.13
	4	0.047	0.014	< 0.01	0.045	0.014	< 0.01
	5	0.065	0.015	< 0.01	0.063	0.015	< 0.01
	6	0.083	0.016	< 0.01	0.077	0.016	< 0.01
	7	0.092	0.017	< 0.01	0.080	0.018	< 0.01
	8	0.100	0.019	< 0.01	0.079	0.021	< 0.01
	9	0.102	0.021	< 0.01	0.065	0.032	0.03
	≥ 10	0.081	0.022	< 0.01	0.078	0.109	0.47

^a^Part of the Randomised Badger Culling Trial (RBCT) in the south west region with three treatments – reactive proactive or no culling of badgers (survey only)

^b ^Standard Error

#### Multivariable modelling

There were 13,595 samples (level 1), from 6,952 cattle (level 2) in 57 herds (level 3) included in the model of herds with seroprevalence < 10%. There was a significantly lower mean S/P ratio in cattle in restocked herds than in continuously stocked herds, and purchased cattle had a significantly higher S/P ratio than homebred cattle (Table 4). There were no significant correlations between explanatory variables.

**Table 4:** Multi-level model of fixed effects associated with *N. caninum *antibody level (S/P ratio) for the herds with a mean herd seroprevalence < 10% (Dataset B).

**Variable**	**Category**	**No. herds**	**No. cattle**	**No. obs.**	**Coef**	**SE^b^**	**P value**
*Intercept*					0.191	0.025	

Triplet code^a^	Reactive	14	1441	3284			
	Proactive	22	3233	7258	-0.004	0.025	0.87
	Survey	21	2270	5682	0.026	0.029	0.37
Restock	No	48	6106	14284			
	Yes	10	846	1970	-0.090	0.030	< 0.01
Farm location	Area A	12	720	1865			
	Area B	40	5365	12320	-0.034	0.030	0.26
	Area C	6	868	2069	-0.025	0.045	0.59
Replacement cattle	Homebred	54	4611	9188			
	Purchased	59	2341	4416	0.123	0.018	< 0.01

^a^Part of the Randomised Badger Culling Trial (RBCT) in the south west region with three treatments – reactive proactive or no culling of badgers (survey only)

^b ^Standard Error

Herd variance 0.002, (s.e. 0.001)

Cattle variance 0.196 (s.e. 0.004)

Visit variance 0.075, (s.e. 0.001)

There were 10,102 samples (level 1), from 4,240 cattle (level 2) in 52 herds (level 3) included in the final model which included herds with a seroprevalence ≥ 10%. There was a significant difference in mean S/P ratio between two year old cattle and all ages above four years (Table 5).

**Table 5:** Multi-level model of fixed effects associated with *N. caninum *antibody level (S/P ratio) for the herds with a mean herd seroprevalence ≥ 10% (Dataset B).

**Variable**	**Category**	**No. herds**	**No. cattle**	**No. obs.**	**Coef**	**SE**^b^	**P value**
*Intercept*					0.441	0.056	

Triplet code^a^	Reactive	24	1885	4689			
	Proactive	12	1433	2990	1.105	0.127	< 0.01
	Survey	16	917	2423	0.035	0.121	0.77
Restock	No	40	3151	7673			
	Yes	14	1090	2510	-0.110	0.072	0.13
Farm location	Area A	24	3651	4285			
	Area B	25	3985	5263	-0.129	0.115	0.26
	Area C	5	255	635	-0.069	0.162	0.67
Age (years)	2	44	699	760			
	3	51	1432	1618	0.054	0.031	0.08
	4	53	1583	1850	0.078	0.032	0.01
	5	50	1272	1450	0.143	0.034	< 0.01
	6	51	1120	1296	0.162	0.035	< 0.01
	7	46	793	1045	0.195	0.037	< 0.01
	8	39	383	773	0.178	0.040	< 0.01
	9	25	135	545	0.201	0.044	< 0.01
	≥ 10	8	16	846	0.160	0.045	< 0.01

^a^Part of the Randomised Badger Culling Trial (RBCT) in the south west region with three treatments – reactive proactive or no culling of badgers (survey only)

^b ^Standard Error

Herd variance 0.004, (s.e. 0.010)

Cattle variance 0.625 (s.e. 0.015)

Visit variance 0.248, (s.e. 0.005)

The greatest unexplained variance in S/P ratios in the final multi-level models for low seroprevalence herds was between cattle (Tables 4 and 5) but all three levels were significant, indicating unexplained variation.

There were two and eight herds that did not have confidence intervals including zero in the model for seroprevalence of < 10% and ≥ 10% respectively in the model fit. Therefore there was a minor violation of the assumption of normality of error terms. These herds departed from the overall average line predicted by the fixed parameters in the final model. However, the removal of any herd, cattle, or sample from the models did not affect the overall interpretation of the results. Three of the four farms with high herd level residuals had a mean seroprevalence > 40% and have been previously discussed. The other herd that also had a relatively high mean seroprevalence (29%) was a dairy herd that continuously stocked through FMD and had an average herd size of 148 cattle.

When the outcome was considered as a binary variable (seropositive/seronegative) and mixed effects logistic binomial regression models were run, the same variables were significant in both models.

## Discussion

Our unique dataset consisted of 114 herds with up to three samples collected at approximately yearly intervals from 15,736 cattle. Nine herds and 4.8% of cattle changed serological status indicating the consistency of *N. caninum *antibody status over the four year study. It is generally assumed that cattle remain permanently infected with *N. caninum*, consequently the cattle that tested seropositive then seronegative and then seropositive could have had false negative results and the test sensitivity was in fact below 100.0%, or some of these cattle might have been incorrectly identified. Previous studies on 18 cows from one herd, 254 cows from one herd and 113 cows from 11 dairy herds have suggested that antibody levels change during pregnancy [13,33,34]. In the current study, this had little effect visually and all cattle seropositive on at least one occasion had significantly higher S/P ratio than seronegative cattle whenever they were sampled during pregnancy. However, cows were not sampled repeatedly through their pregnancy, and therefore these data cannot not be compared with previous results [13,33,34]. This does not indicate that changing values in pregnancy are not important to understand disease pathogenesis, but for the purposes of this study they are unlikely to have influenced the results. However, the previous reports of 87.4% of seropositive cows staying seropositive throughout pregnancy [33], and 2 out of 30 seropositive cows and 1 out of 83 seronegative cows changing their serological status during pregnancy [34] may further explain why for a few cattle serostatus changed once or twice. Overall, the consistency of the serological status suggest that the reported sensitivity and specificity of the kit were reasonably accurate with little bias and misclassification. A number of cows had no date of birth in the BCMS. The exclusion of cattle with missing data did not appear to affect the results of this study.

In the current study, the large sample size of 114 herds enabled us to categorise herds into high and low seroprevalence for *N. caninum *and the prospective nature of the data enabled us to investigate seroconversion/recrudescence. This was a useful strategy since the greatest risk from an infectious disease is usually close contact conspecifics; investigating high and low prevalence herds divided at the median permitted examination of a within herd exposure to *N. caninum*. Despite this, there was no association between S/P ratio and herd size. This has been reported in some studies [35,36] but not others [23], indicating that there is as yet no consensus on this link.

There was, however, an association between seroprevalence and age. This is in contrast to results from a smaller study of 14 Great Britain herds [23], where the prevalence of *N. caninum *antibody in 7–12 month old cattle was not significantly different from the prevalence in older cattle. In the current study, the association between seroprevalence and age was only present in herds with a mean seroprevalence ≥ 10% and not in herds with a mean seroprevalence < 10%. The increased seroprevalence with age (Figure 3) indicates either horizontal transmission in high seropositive herds or the selective culling of seropositive 2 and 3 years old cattle (that leads to a reduced seroprevalence in these two age groups) [28]. This may indicate that there are no important clinical signs in seropositive cattle, or that the prevalence of infected cattle was too high to remove them all without raising culling rates to an uneconomically high level [37].

The antibody prevalence to *N. caninum *was not, as with many viral infections, monotonically increasing with age, but plateaued at about 4 years of age. This could be due either to a waning immunity and intermittent exposure (i.e. if cattle are not re-exposed to *N. caninum *their antibody level falls, and becomes negative), or to reduced survival (i.e. individuals that are *N. caninum *antibody positive are less likely to survive). We believe that the most consistent explanation for the age-related seroprevalence patterns is increased horizontal transmission in high prevalence herds, but with non-continuous exposure.

In our study there were indications that either horizontal transmission or recrudescence occurred in cattle in herds with a mean seroprevalence ≥ 10%: some of these cattle were purchased seronegative cattle that seroconverted (7.2% of the purchased seronegative cattle later seroconverted) and some were homebred offspring with seronegative dams that were seropositive themselves (5.1% of the dam-calf pairings the dam was seronegative but the calf was seropositive). Further evidence for this is that the 10 restocked herds of cattle in herds with a median seroprevalence < 10% had a lower S/P ratio within the herds but cattle from the 14 restocked herds with a median seroprevalence ≥ 10% did not have a lower S/P ratio. This suggests that there were restocked herds where *N. caninum *infection had not spread and others where *N. caninum *infection had spread in the time since restocking.

These results are consistent with studies that have suggested that vertical transmission may be dominant but that horizontal transmission must occur for the parasite to persist [18,20]. It has been proposed elsewhere that the probability of horizontal transmission increases as the herd seroprevalence increases [21]. Seropositive cattle in the herd may create a positive feedback for infection from horizontal and vertical transmission of *N. caninum*, aiding persistence. Unfortunately, this study cannot determine how these cattle became infected. Other studies have suggested that dogs, red foxes, sheep, goats, horses and presumably cattle tissues may be sources of infection [9,10].

Indicators for vertical transmission, reported in many other studies [12,18] were also present in the current study. There was a significant positive association in serological status between dams and daughters in all herds and this, and purchased cattle, were the only statistical associated risks for seropositive cattle in herds with a mean seroprevalence < 10%. Both dam and herd effect were apparent, but the dam effect was greater because there was a higher proportion of positive calves born from seropositive dams in low seroprevalence herds than calves born from seronegative dams in high seroprevalence herds.

The mean *N. caninum *S/P ratio was higher in purchased cattle than homebred cattle in herds with mean seroprevalence < 10%. It is intuitive that the likelihood of purchasing a cow with a higher S/P ratio than the herd mean is high when the herd seroprevalence is low. Consequently, purchasing cattle may increase the seroprevalence of *N. caninum *antibody in herds with low seroprevalence. This movement of cattle may ultimately reduce between-herd variability in seroprevalence to *N. caninum *as introduction of infected cattle into naïve herds and reintroduction into infected herds occurs. It also aids persistence of the parasite in the population [29], assuming that infected cows are, at least sometimes, infectious. This is already reflected in the results with only 6% herds with no seropositive cattle, and in the small value of the unexplained between-herd variance of *N. caninum *S/P values in the multivariable models. Our results also highlight the practical importance of testing purchased cattle before introducing them to a naïve or low *N. caninum *seropositive herd [27,28].

In a country where purchasing occurs more uniformly than in Great Britain one would expect the between-herd variability in seroprevalence to be lower, and that ultimately there may be no difference in *N. caninum *antibody seroprevalence between herds and consequently between purchased and homebred cattle (i.e. homogenous mixing). This may explain the results from Sanderson et al. (2000) from Northwest United States who studied 2,585 cows in 55 beef herds and reported that there was no association between cow origin and serostatus to *N. caninum *[38].

## Conclusion

*N. caninum *antibodies were widespread in these 114 herds and the seroprevalence between herds was variable but consistent over the four year study indicating that large changes in *N. caninum *antibody across herds are temporally slow. In all herds there was evidence of vertical transmission of *N. caninum*. In herds with a mean seroprevalence < 10% there was evidence for introduction of infection through purchased cattle; there was no evidence for horizontal transmission with no change in seroprevalence with cattle age and no evidence that seronegative mothers were associated to seropositive offspring. In contrast, there was evidence for horizontal transmission and/or recrudescence of infection in herds with a mean seroprevalence ≥ 10%. Seroprevalence increased in cattle between 2 and 4 years of age and offspring of seronegative dams were likely to become seropositive with age. This indicates a positive feedback for infection in these high prevalence herds and the possibility that two states may arise, herds with a stable low seroprevalence and herds with a stable high seroprevalence with different within herd dynamics.

## Authors' contributions

KAW helped collect samples, performed the statistical analysis and drafted the manuscript. LEG participated in the design of the study, data collection and data analysis, was co-applicant for funding, and helped to draft the manuscript. GFM participated in the design of the study and data analysis and was co-applicant for funding. SJM coordinated ELISA testing. ARV coordinated data collection and helped collect samples. SM setup and maintained the study database. All authors read and approved the final manuscript.

## References

[B1] Davison HC, Otter A, Trees AJ (1999). Significance of *Neospora caninum *in British dairy cattle determined by estimation of seroprevalence in normally calving cattle and aborting cattle. Int J Parasitol.

[B2] Hobson JC, Duffield TF, Kelton D, Lissemore K, Hietala SK, Leslie KE, McEwen B, Cramer G, Peregrine AS (2002). Neospora caninum serostatus and milk production of Holstein cattle. J Am Vet Med Assoc.

[B3] Barling KS, McNeill JW, Thompson JA (2000). Association of serologic status for *Neospora caninum *with postweaning weight gain and carcass measurements in beef calves. J Am Vet Med Assoc.

[B4] Thurmond MC, Hietala SK (1996). Culling associated with *Neospora caninum *infection in dairy cows. Am J Vet Res.

[B5] Thurmond MC, Hietala SK (1997). Effect of congenitally acquired *Neospora caninum *infection on risk of abortion and subsequent abortions in dairy cattle. Am J Vet Res.

[B6] Trees AJ, Davison HC, Innes EA, Wastling JM (1999). Towards evaluating the economic impact of bovine neosporosis. Int J Parasitol.

[B7] McAllister MM, Dubey JP, Lindsay DS, Jolley WR, Wills RA, McGuire AM (1998). Dogs are definitive hosts of Neospora caninum. Int J Parasitol.

[B8] Gondim LF, McAllister MM, Pitt WC, Zemlicka DE (2004). Coyotes (Canis latrans) are definitive hosts of *Neospora caninum*. Int J Parasitol.

[B9] Moore DP (2005). Neosporosis in South America. Vet Parasitol.

[B10] Gondim LF (2006). *Neospora caninum *in wildlife. Trends Parasitol.

[B11] Dubey JP (1999). Recent advances in Neospora and neosporosis. Vet Parasitol.

[B12] Björkman C, Johansson O, Stenlund S, Holmdahl OJ, Uggla A (1996). Neospora species infection in a herd of dairy cattle. J Am Vet Med Assoc.

[B13] Stenlund S, Kindahl H, Magnusson U, Uggla A, Björkman C (1999). Serum antibody profile and reproductive performance during two consecutive pregnancies of cows naturally infected with *Neospora caninum*. Vet Parasitol.

[B14] Innes EA, Wright S, Bartley P, Maley S, Macaldowie C, Esteban-Redondo I, Buxton D (2005). The host-parasite relationship in bovine neosporosis. Vet Immunol Immunopathol.

[B15] Anderson ML, Reynolds JP, Rowe JD, Sverlow KW, Packham AE, Barr BC, Conrad PA (1997). Evidence of vertical transmission of Neospora sp infection in dairy cattle. J Am Vet Med Assoc.

[B16] Frössling J, Uggla A, Björkman C (2005). Prevalence and transmission of *Neospora caninum *within infected Swedish dairy herds. Vet Parasitol.

[B17] Hall CA, Reichel MP, Ellis JT (2005). Neospora abortions in dairy cattle: diagnosis mode of transmission and control. Vet Parasitol.

[B18] Davison HC, Otter A, Trees AJ (1999). Estimation of vertical and horizontal transmission parameters of *Neospora caninum *infections in dairy cattle. Int J Parasitol.

[B19] French NP, Clancy D, Davison HC, Trees AJ (1999). Mathematical models of *Neospora caninum *infection in dairy cattle: transmission and options for control. Int J Parasitol.

[B20] Hietala SK, Thurmond MC (1999). Postnatal *Neospora caninum *transmission and transient serologic responses in two dairies. Int J Parasitol.

[B21] Romero JJ, Frankena K (2003). The effect of the dam-calf relationship on serostatus to *Neospora caninum *on 20 Costa Rican dairy farms. Vet Parasitol.

[B22] Wapenaar W, Barkema HW, Vanleeuwen JA, McClure JT, O'Handley RM, Kwok OC, Thulliez P, Dubey JP, Jenkins MC (2007). Comparison of serological methods for the diagnosis of Neospora caninum infection in cattle. Vet Parasitol.

[B23] Davison HC, French NP, Trees AJ (1999). Herd-specific and age-specific seroprevalence of *Neospora caninum *in 14 British dairy herds. Vet Rec.

[B24] McNamee PT, Trees AJ, Guy F, Moffett D, Kilpatrick D (1996). Diagnosis and prevalence of neosporosis in cattle in Northern Ireland. Vet Rec.

[B25] Trees AJ, Guy F, Low JC, Roberts L, Buxton D, Dubey JP (1994). Serological evidence implicating Neospora species as a cause of abortion in British cattle. Vet Rec.

[B26] Bartels CJ, Arnaiz-Seco JI, Ruiz-Santa-Quitera A, Björkman C, Frössling J, von Blumröder D, Conraths FJ, Schares G, van Maanen C, Wouda W, Ortega-Mora LM (2006). Supranational comparison of *Neospora caninum *seroprevalences in cattle in Germany The Netherlands Spain and Sweden. Vet Parasitol.

[B27] Williams DJ, Trees AJ (2006). Protecting babies: vaccine strategies to prevent foetopathy in Neospora caninum-infected cattle. Parasite Immunol.

[B28] Dubey JP, Schares G, Ortega-Mora LM (2007). Epidemiology and control of neosporosis and *Neospora caninum*. Clin Microbiol Rev.

[B29] Bourne J (2007). Bovine TB: The Scientific Evidence. A science base for a sustainable policy to control TB in cattle. An epidemiological investigation into bovine tuberculosis. Defra Final Report.

[B30] Keeling MJ, Woolhouse ME, May RM, Davies G, Grenfell BT (2003). Modelling vaccination strategies against foot-and-mouth disease. Nature.

[B31] von Blumröder D, Schares G, Norton R, Williams DJ, Esteban-Redondo I, Wright S, Björkman C, Frössling J, Risco-Castillo V, Fernández-García A, Ortega-Mora LM, Sager H, Hemphill A, van Maanen C, Wouda W, Conraths FJ (2004). Comparison and standardisation of serological methods for the diagnosis of *Neospora caninum *infection in bovines. Vet Parasitol.

[B32] Rasbash J, Browne W, Goldstein H, Yang M, Plewis I, Healy M, Woodhouse G, Draper D, Langford I, Lewis T (2000). A user's guide to *MLwiN *version 21d for use with *MLwiN *1.10. Centre for Multilevel Modelling Institute of Education University of London.

[B33] Stenlund S, Kindahl H, Magnusson U, Uggla A, Björkman C (1999). Serum antibody profile and reproductive performance during two consecutive pregnancies of cows naturally infected with Neospora caninum. Vet Parasitol.

[B34] Häsler B, Hernandez JA, Reist M, Sager H, Steiner-Moret C, Staubli D, Stärk KDC, Gottstein B (2006). Neospora caninum: Serological follow-up in dairy cows during pregnancy. Vet Parasitol.

[B35] Otranto D, Llazari A, Testini G, Traversa D, Frangipane di Regalbono A, Badan M, Capelli G (2003). Seroprevalence and associated risk factors of neosporosis in beef and dairy cattle in Italy. Vet Parasitol.

[B36] Kyaw T, Virakul P, Muangyai M, Suwimonteerabutr J (2004). Neospora caninum seroprevalence in dairy cattle in central Thailand. Vet Parasitol.

[B37] Green LE (2007). Improving farm animal health – understanding infectious endemic disease. Proceedings of the SVEPM conference: March 2007; Finland.

[B38] Sanderson MW, Gay JM, Baszler TV (2000). *Neospora caninum *seroprevalence and associated risk factors in beef cattle in the northwestern United States. Vet Parasitol.

